# First-principle study of structural, electronic and magnetic properties of (FeC)_n_ (n = 1–8) and (FeC)_8_TM (TM = V, Cr, Mn and Co) clusters

**DOI:** 10.1038/s41598-017-17834-9

**Published:** 2017-12-13

**Authors:** Cheng-Gang Li, Jie Zhang, Wu-Qin Zhang, Ya-Nan Tang, Bao-Zeng Ren, Yan-Fei Hu

**Affiliations:** 1 College of Physics and Electronic Engineering, Quantum Materials Research Center, Zhengzhou Normal University, Zhengzhou, 450044 China; 2 School of Chemical Engineering and Energy, Zhengzhou University, Zhengzhou, 450001 China; 3 School of Physics and Electronic Engineering, Sichuan University of Science & Engineering, Zigong, 643000 China

## Abstract

The structural, electronic and magnetic properties of the (FeC)_n_ (n = 1–8) clusters are studied using the unbiased CALYPSO structure search method and density functional theory. A combination of the PBE functional and 6–311 + G* basis set is used for determining global minima on potential energy surfaces of (FeC)_n_ clusters. Relatively stabilities are analyzed via computing their binding energies, second order difference and HOMO-LUMO gaps. In addition, the origin of magnetic properties, spin density and density of states are discussed in detail, respectively. At last, based on the same computational method, the structures, magnetic properties and density of states are systemically investigated for the 3*d* (V, Cr, Mn and Co) atom doped (FeC)_8_ cluster.

## Introduction

Magnetic clusters are aggregates of a few to thousands of atoms or molecular that exhibit magnetism. Since the foundation of quantum mechanics, the magnetism has been extensively investigated and well understood. Many workers have witnessed increasing interest in the magnetic properties of clusters from both basic science and technological applications^1^. The magnetic behaviors of a cluster can be measured by the Stern-Gerlach deflection and XMCD (X-ray magnetic circular dichroism spectroscopy) in a molecular beam. With the rapid progress in computational physics, now it is possible to study magnetic properties of cluster from first-principles calculations, and get more and accurate information. Due to partly filled *d* shells and an accompanying complicated energy landscape, the transition-metal (TM) clusters show a different magnetic behavior from that of the corresponding bulk, and have attracted resurgent interests in new magnetic materials^2–4^. For example, enhanced magnetic moments have been observed in small clusters of iron and cobalt^5–7^, and ferromagnetic or ferromagnetic ordering has been identified in chromium (Cr_8_-Cr_156_) and manganese (Mn_5_-Mn_99_) cluster, even though Cr and Mn are both antiferromagnetic in the bulk phase^5,8^. Besides, many experimental investigations have been employed to study the magnetic properties of Ni_n_, Co_n_ and Fe_n_ clusters^9–14^. For example, Cox *et al*.^9^ performed the first measurement of Stern-Gerlach deflection of individual mass-selected Fe_n_ clusters, and found that these clusters are paramagnetic, possessing magnetic moments which increase linearly with cluster size. Bloomfield’s group^10–12^ carried out a series of Stern-Gerlach measurements on the magnetic properties of Co and Ni clusters. Peredkov *et al*.^13^ measured temperature dependent XMCD of Co_n_ clusters, from which the intrinsic spin and orbital magnetic moments are deduced. In theoretical factor, Bhunia *et al*.^15^ performed a comprehensive DFT (density functional theory) calculation of the magnetic properties of Sc_n_ (n = 2–14) clusters. The magnetic moments of Cr_7_, Mn_7_ and Fe_7_ clusters are calculated by the discrete variational non-collinear spin-density functional method^16^. In addition, several *ab initio* calculations are carried out to understand the structural, electronic and magnetic properties of Mn, Cr and Co clusters^17–19^. To date, the rich and varied magnetic behavior displayed in bulk binary alloys suggests that bimetallic TM clusters may display interesting and potentially useful magnetic properties. Among the 30 kinds of TM clusters, due to the position of iron between the early and late TM, carbon-iron clusters have obtained comprehensive understanding of the geometrical properties, electron structures and magnetism regardless of the experimental measurements or theoretical calculations^20–27^. For example, FeC molecules are generated in a laser vaporization molecular beam source and detected by laser induced fluorescence^20^. Based on the photoelectron spectroscopy, the electronic structure and chemical bonding are studied for FeC_2_^−^ clusters by Li *et al*.^21^. Fan and co-workers^22^ measured the photoelectron spectra of FeC_3_^−^ and FeC_4_^−^ clusters at 3.49 eV photon energy. Later, several data such as the bond lengths, ionization and dissociation energies, vibrational frequencies and dipole moment are also determined by experimental measurements^23–28^. A large number of theoretical reports are available for describing the effects of size and structure to change the electronic and other properties of clusters. The geometric conformations and electronic states of FeC_2_ cluster are studied by Arbuznikov *et al*.^29^ using the DFT (B3LYP) and CASSCF/CASPT2 methods. On the basis of the DMol3 package, Ryzhkov *et al*.^30^ carried out an extensive study for the geometric structures, effective charges and total spin densities of Fe_2_C, FeC_2_, Fe_3_C, FeC_3_ and Fe_2_C_2_ clusters. From this study it reflects that the triangular configurations of FeC_2_ and Fe_2_C contain the lowest binding energy. The most stable planar structures for FeC_3_ and Fe_2_C_2_ are favored over the three-dimensional isomers. Recently, Wang *et al*.^31^ reported photoelectron spectra of FeC_3_^−^ cluster, and found that the ground states of FeC_3_ and FeC_3_^−^ clusters have *C*_2v_ ring structures. Using gas phase ion chromatography, Helden *et al*.^32^ studied the structures of Fe_n_C_m_ (n = 1–3 and m = 2–8) clusters, and observed different chemical compositions possess the different structure. Practically speaking, clusters with one iron atom have linear structures for m ≤4, while larger clusters exhibited cyclic structures. Clusters with two iron atoms are pure monocyclic rings. The three iron species possess three-dimensional structures. Very recently, Ma’s group^33^ investigated the structures and magnetic moments of FeC_n_ (n = 1–8) and Fe_2_C_n_ (n = 1–6) clusters by all-electron density functional theory. Results showed that the ground states of FeC_n_ (n = 1–8) clusters have the linear structures with the Fe atom bonded at one end, except for FeC_2_. Fe_2_C_n_ clusters with even *n*, plus Fe_2_C, have cyclic planar structures with transannular bonds. Fe_2_C_n_ clusters with odd n (n = 3, 5) prefer linear geometries with the two Fe atoms at the two ends. Furthermore, analysis of the Mulliken population showed charge transfers from the Fe atoms to the C atoms with the magnetic moment lying primarily on the Fe atoms. Although a number of experimental and theoretical studies have been done on the structural, electronic and magnetic properties of Fe_n_C_m_ clusters, a theoretical analysis of the structures, electronic and magnetic properties is still unexplored for (FeC)_n_ (n = 1–8) clusters. Since, the study of the cluster size structure is a prerequisite for understanding their electronic and magnetic properties. The determination of lowest energy structure plays a crucial role. However, because of much increased complexity of the potential surface as well as the exponential increase of structures with increasing number of atoms, the determination of the true global minimum structure is a challenging problem.

In the present work, we carried out an extensive structure search for (FeC)_n_ (n = 1–8) clusters by combining the Crystal structure Analysis by Particle Swarm Optimization (CALYPSO) searching method and density functional theory calculations^34–36^. This combined CALYPSO/DFT computational approach has been previously used to search for low-lying palladium clusters, copper clusters, ruthenium doped germanium clusters *et al*.^37–40^. Based on the lowest energy structure, the electronic and magnetic properties are investigated in detail as the cluster size increase. At last, the structures, magnetic properties and density of states are systemically investigated with the same computational method for the 3*d* (V, Cr, Mn and Co) atom doped (FeC)_8_ cluster.

The paper is organized as follows: we present and discuss the findings pertaining to geometrical structures, relative stability, magnetic properties and density of states of the ground state structures in the following. Subsequently, the structures, magnetic properties and density of states are systemically investigated for (FeC)_8_TM (TM = V, Cr, Mn and Co) clusters. Then, our conclusions are summarized. Finally, details of the computational method are concisely described.

## Results and Discussions

### Structures of (FeC)_n_ (n = 1–8) clusters

**Figure 1:**
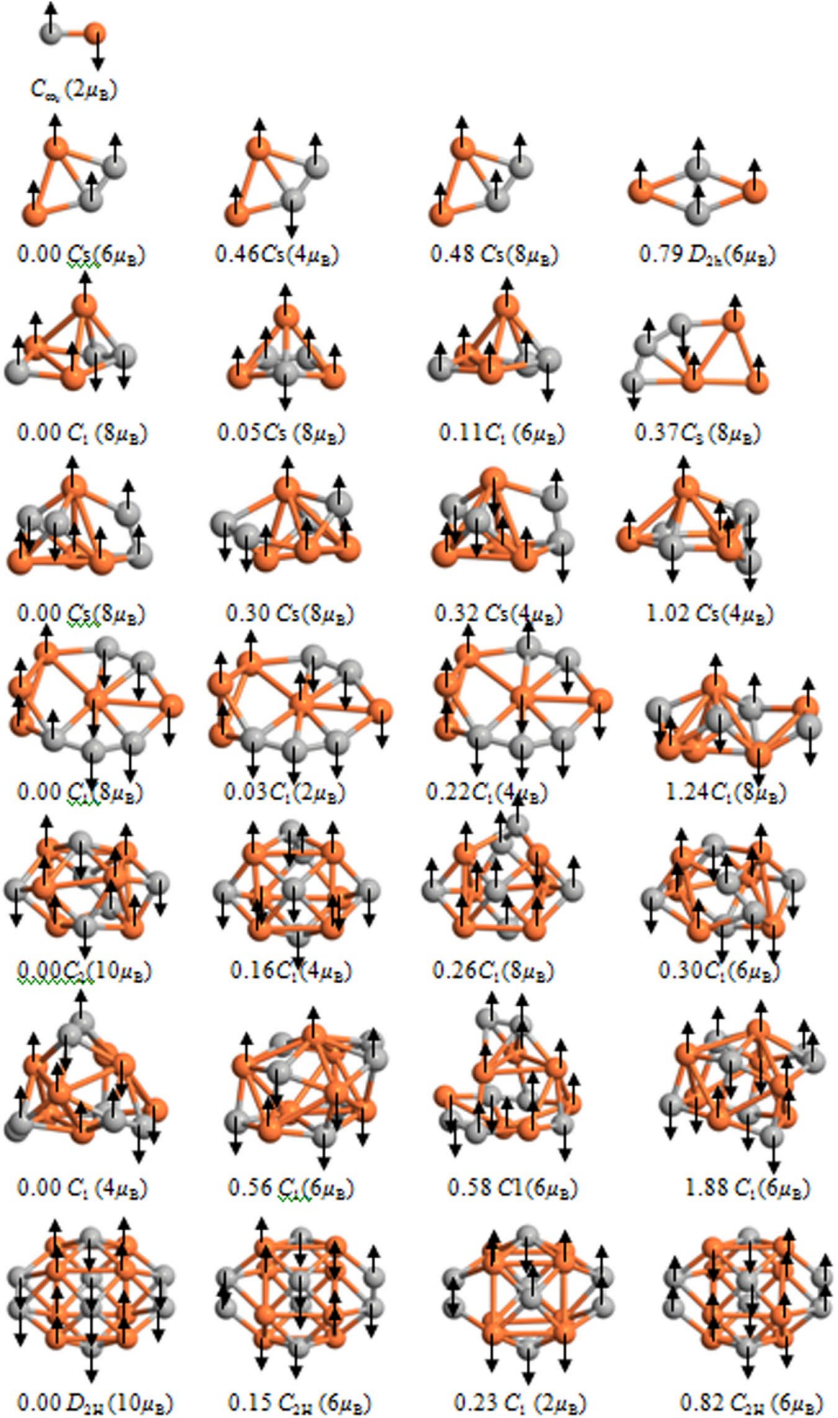
The lowest energy and some metastable structures of (FeC)_n_ (n = 1–8) clusters. Orange ball: Fe atom; gray ball: C atom. Values out and in parentheses indicate the relative energies (eV) and total spin magnetic moment (*μ*_B_). The spin orientation of Fe and C atoms is labeled.

The global minimum structures of the (FeC)_n_ (n = 1–8) clusters are shown in Fig. 1 along with their three low-lying isomers for discussion, in which relative energies, point group symmetry and total spin magnetic moments are also presented in Fig. 1. The corresponding Cartesian coordinates and the local and total spin magnetic moments are calculated and listed in Table S1 in the Supporting Information (SI).

The lowest energy structure of FeC (*C*_∞*v*_) with bond length 1.56 Å, which is smaller than the Fe-Fe bond (2.01 Å) and bigger than the C-C bond (1.32 Å), possesses ferrimagnetic ordering and a total spin magnetic moment of 2 *μ*_B_ (considering spin orientation).

For (FeC)_2_ clusters, a distorted parallelogram (*C*_s_) is found to be the ground state, of which the four atoms have ferromagnetic ordering corresponding to a total spin magnetic moment of 6 *μ*_B_ and an average spin moment per Fe atom of 2.84 *μ*_B_ (without considering spin orientation). The second isomer (*C*_s_) with a ferrimagnetic C-C coupling and ferromagnetic Fe-Fe coupling is 0.46 eV higher in energy than the ground state. The third isomer (*C*_s_) with the same spin orientation has a total moment 8 *μ*_B_ and is 0.48 eV higher than the ground state. The rhombuses structure (*D*_2H_) is 0.79 eV higher in energy than the ground state with Fe and C atoms located at opposite vertices. The structure has ferromagnetic ordering and a total spin magnetic moment of 6 *μ*_B_. In conclusion, all of atoms of 1st, 3rd and 4th structures have ferromagnetic ordering; while the C-C coupling in the second structure possesses ferromagnetic ordering. So, the second structure possesses the smallest total spin moment of 4 *μ*_B_.

From n = 3 on, the ground state (*C*_1_) is found to be three-dimensional structure with a total spin magnetic moment 8 *μ*_B_ and a mean spin moment per Fe atom of 2.7 *μ*_B_. Two other low-lying isomers (*C*_s_-2, *C*_1_-3) are 0.05 eV and 0.11 eV higher in energy than the ground state. The plan pentagonal structure with total spin moment 8 *μ*_B_ lies only 0.37 eV higher in energy. It is worth mentioning that all of Fe-Fe coupling of the structures is ferromagnetic ordering.

For (FeC)_4_, the *C*_s_-1 structure is obtained as the lowest energy structure, having a total spin magnetic moment of 8 *μ*_B_ and the mean spin moment per Fe atom of 2.08 *μ*_B_. The *C*_s_ structure has energy 0.30 eV higher than that of the ground state and is found to be the second isomer. Two other low-lying isomers have *C*_s_ structure and lie 0.32 eV, 1.02 eV, respectively, higher in energy.

In the case of (FeC)_5_, the total spin magnetic moment is 8 *μ*_B_ for the ground state (*C*_1_). The 2nd and 3rd have the same symmetry structure as like of the ground state. And, total spin magnetic moments are 2 *μ*_B_ and 4 *μ*_B_, respectively. The fourth structure (*C*_1_) with total spin magnetic moments are 8 *μ*_B_ is 1.24 eV higher in energy than the ground state.

At n = 6, the cage type (*C*_2_) structure is found to be the ground state structure. The corresponding total spin magnetic moment and mean spin moment per Fe atom are 10 *μ*_B_ and 1.76 *μ*_B_, respectively. This is followed by three low-lying isomers (*C*_1_-2, *C*_1_-3, *C*_1_-4). And, the relative energies compared with the ground state are 0.16 eV, 0.26 eV and 0.30 eV, respectively.

With regard to (FeC)_7_, the *C*_1_-1 structure is obtained as the ground state structure having a total spin magnetic moment of 4 *μ*_B_. The *C*_1_ structure has energy 0.56 eV higher than that of the ground state and is found to be the second isomer. The *C*_1_-3 and *C*_1_-4 structures have total spin magnetic moments 6 *μ*_B_ and 6 *μ*_B_, respectively, and they lie 0.58 eV and 1.88 eV higher than the ground state.

For (FeC)_8_, the total spin magnetic moment and mean spin moment per Fe atom of the lowest energy structure (*D*_2H_) are 10 *μ*_B_ and 1.34 *μ*_B_, respectively. The structure with *C*_2H_-2, *C*_1_-3, *C*_2H_-4, respectively, are all with Fe and C atoms locating at the surface sites and is 0.15 eV, 0.23 eV and 0.82 eV higher in energy than the ground state. The corresponding total spin magnetic moments are 6 *μ*_B_, 2 *μ*_B_ and 6 *μ*_B_, respectively.

To validate the effectiveness of functional and basis set, a comparative analysis is made between experimental spin magnetic moments, bond length and our computed data of the species namely C_2_, Fe_2_, FeC and (FeC)_2_. For example, the calculated total spin magnetic moments (2 *μ*_B_, 6 *μ*_B_ and 2 *μ*_B_) for C_2_, Fe_2_ and FeC clusters are in good agreement with the theoretical values (2 *μ*_B_, 6 *μ*_B_ and 2 *μ*_B_)^41,42^. For FeC clusters, the present bond length (1.563 Å) is consistent with those previous values (1.61 Å and 1.575 Å) obtained by Ma and Noya *et al*. using DFT and DMOL package^33,43^. For (FeC)_2_ cluster, the calculated total spin magnetic moment (6 *μ*_B_) is excellent agreement with Ma *et. al*’s work (6 *μ*_B_)^33^. The overall good agreement between the experiment and theory lends good confidence for the selected levels of theory used for other more complicated iron-carbon species in the current study.

### Relatively stabilities and electronic properties

**Figure 2:**
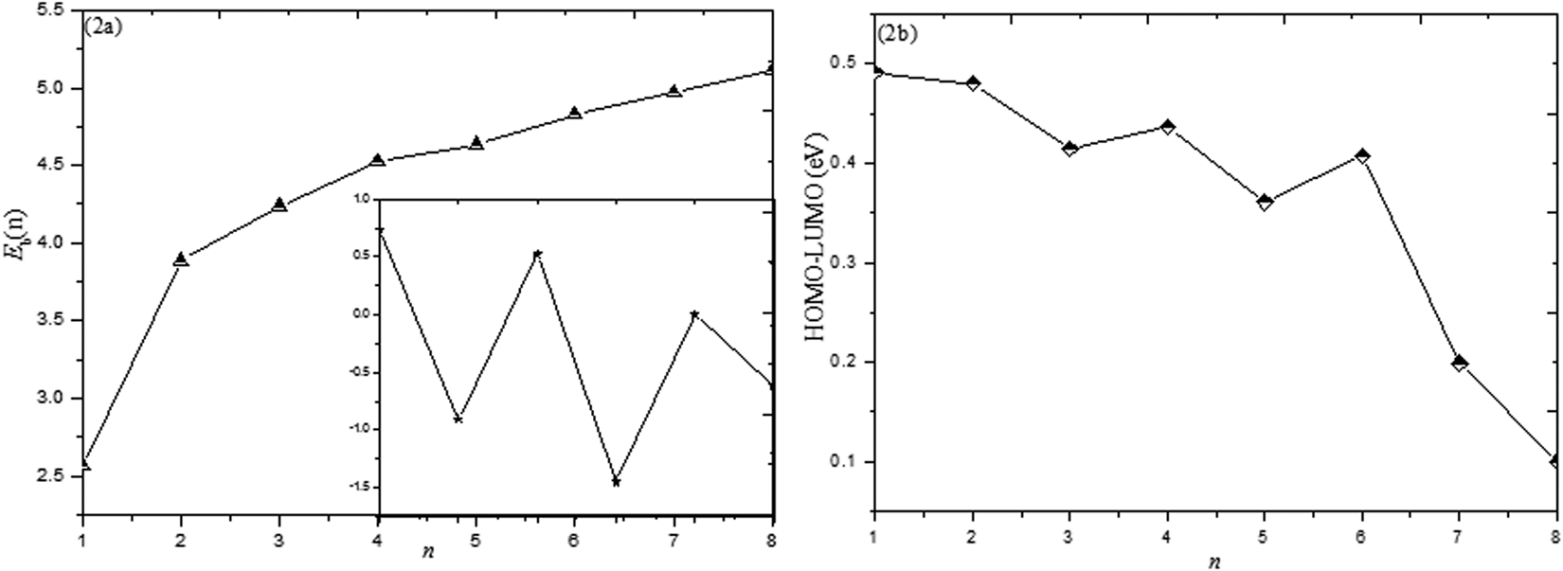
The variation of binding energy and HOMO-LUMO corresponding to the most stable geometry; the chart in the inset is the second order difference (all units in eV).

Stability of a cluster could be judged through its binding energy $E{}_{b}(n)$ per atom, which can be defined as:

1 $${E}_{b}(n)=[nE({\rm{F}}{\rm{e}})+nE(C)-E{({\rm{F}}{\rm{e}}{\rm{C}})}_{n}]/2n$$

where *E*(Fe), *E*(C), *E*(FeC)_n_ are an isolated Fe atom, an isolated C atom and the total energies of (FeC)_n_ clusters, respectively. The $E{}_{b}(n)$ quantities as a function of cluster size are given in Fig. 2a. Significantly, we can see that the *E*_b_(n) values increase monotonically with cluster size, revealing that the stabilities are relatively enhanced. Moreover, the values of *E*_*b*_(n) increases rapidly in the size range from 1 to 2.

The second order difference of energy ∆_2_ *E*(n) is a sensitive quality, reflecting the relatively stability of clusters. In cluster physics, ∆_2_ *E*(n) is defined as:

2 $${{\rm{\Delta }}}_{2}E(n)=E{({\rm{F}}{\rm{e}}{\rm{C}})}_{n-1}+E{({\rm{F}}{\rm{e}}{\rm{C}})}_{n+1}-2E{({\rm{F}}{\rm{e}}{\rm{C}})}_{n}$$

*E*(FeC)_n_, *E*(FeC)_n−1_ and *E*(FeC)_n+1_ represent the total energies of the corresponding clusters, respectively. The plot between second order energy difference and cluster size is plotted in the inset in Fig. 2a. Quite clearly, three distinct peaks at n = 2, 4 and 6 are observed, which indicate that (FeC)_2_, (FeC)_4_ and (FeC)_6_ are relatively more stable than its neighboring clusters. Remarkably, n = 2 correspond to the largest second order difference of energies.

**Figure 3:**
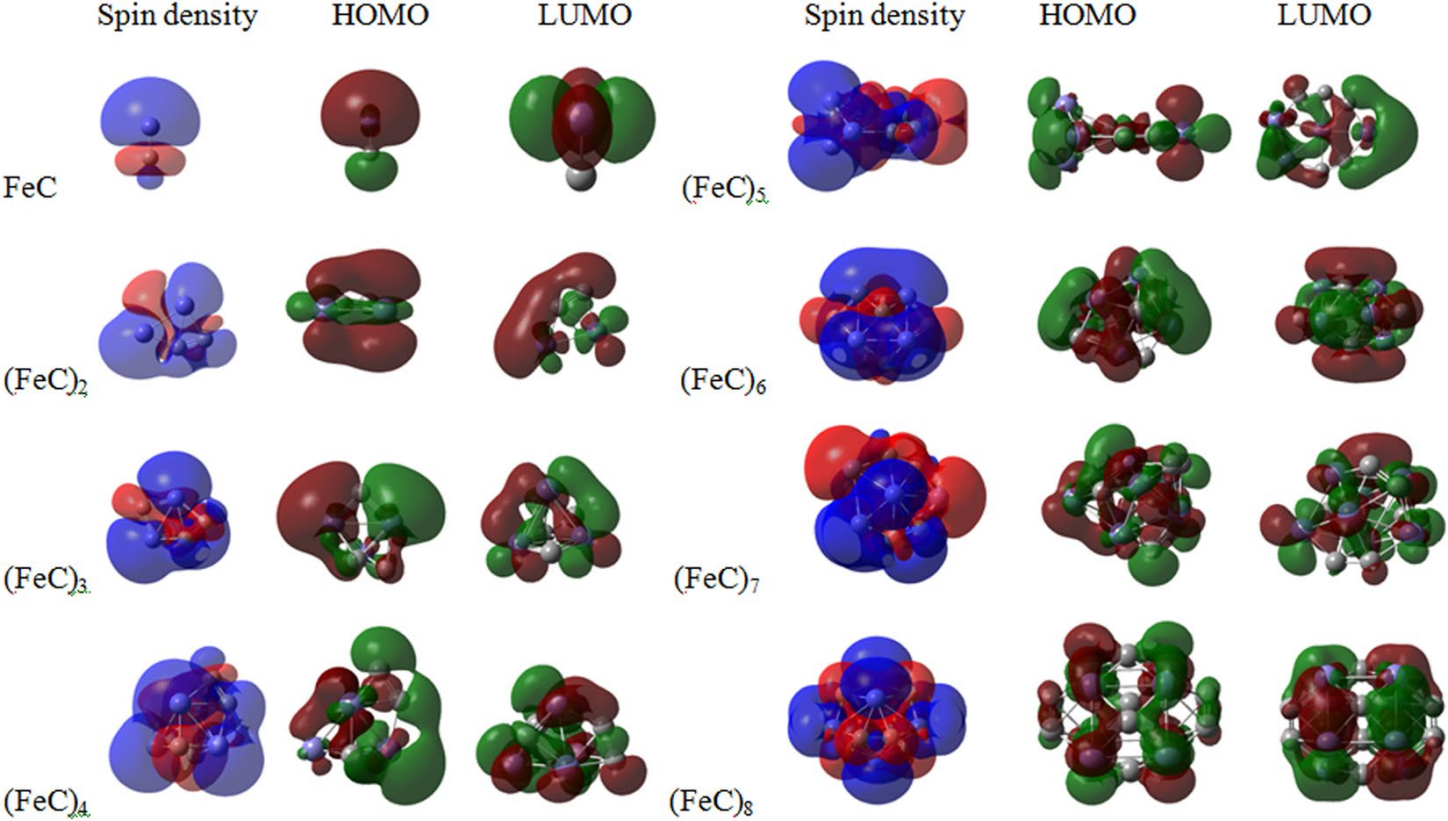
The spin density, HOMO and LUMO isosurfaces of the lowest energy structures of (FeC)_n_ (n = 1–8) clusters.

The energy difference between the highest occupied molecular orbital (HOMO) and lowest unoccupied molecular orbital (LUMO) is considered to be an important symbol in terms of the electronic stability. A large HOMO-LUMO energy gap is characteristic of chemical stability. The evolution of HOMO-LUMO energy gap (HLEG) for the ground state (FeC)_n_ (n = 1–8) clusters is shown in Fig. 2b. Clearly, two local maxima are found at n = 1 and 2, suggesting that FeC and (FeC)_2_ clusters are more stable than their neighbors. For further to determine the stability of clusters, the Mayer bond order are included. Generally, the value of Mayer bond order is in agreement with empirical bond order, for single, double and triple bond the value is close to 1.0, 2.0 and 3.0 respectively. For (FeC)_2_ cluster, the bond order of carbon-carbon bond (1.989) is close to 2.0, implying there exists a double-bond between carbon-carbon bonds. Moreover, the bond order of Fe-Fe bond has a relatively larger value (1.359). So, the stability of (FeC)_2_ is mainly due to the formation of the strong C = C bond and the formation of the Fe-Fe bond. In addition, the calculated HOMO and LUMO isosurface is lucidly plotted in the Fig. 3. In Fig. 3, the HOMOs are mostly localized at Fe atoms, a small part of the orbital of C atoms are surrounded by LUMOs. Comparing the HOMO and LUMO isosurface of different (FeC)_n_ clusters, we can infer that the chemical stability is dependent on the Fe atom in the clusters.

The shell model of metal clusters is based on fully delocalized molecular orbitals (MOs) for the cluster electrons, which the valence electrons of the cluster atoms are placed in shells of *s*, *p*, and *d* character of the overall system^44–47^. Here, for an in-depth understanding the shell model of clusters, we analyzed their MOs based on the Multiwfn program package^48^. As evidenced by the diagram, the HOMO and LUMO of FeC cluster possess Fe-3*d* atomic orbital (99%) and very small C-2*p* orbital. However, for (FeC)_8_ cluster, the LUMO contains Fe-3*d* (60.29%) orbital with admixtures of Fe-4*s* (11.00%) and C-2*p* (19.66%) character; For HOMO, the Fe-3*d* orbital decreased to 57.16% with admixtures of Fe-4*s* (3.68%), Fe-4*p* (4.96%), C-2*p* (26.84%) and C 2 s (4.1%) orbital.

To further elucidate the magnetic behavior of clusters, the spin density of is also shown in Fig. 3. Blue and red represent the excess of spin up and spin down electrons, respectively, which indicates the positive and negative magnetic moments. For FeC cluster, the Fe atom is surrounded completely by the blue region, it implies that Fe atom has larger positive magnetic moments. For C atom, it is encircled by red and blue region. Because of the regions with red area is larger than that of blue area, C atom possesses negative magnetic moments. For (FeC)_8_ cluster, the blue region mainly localizes around the Fe atoms, while there are red region around eight C atoms. It indicates that C atoms possess the contrary magnetic ordering to Fe atoms. Furthermore, the spin density distribution is larger in the Fe atoms than that of C atoms, which indicates that the behavior of Fe atoms will dominate the magnetic behavior. Present analyses agree satisfactorily with those previously discussion based on the local and total spin moment and coupling ordering in Table S1 (see SI).

**Figure 4:**
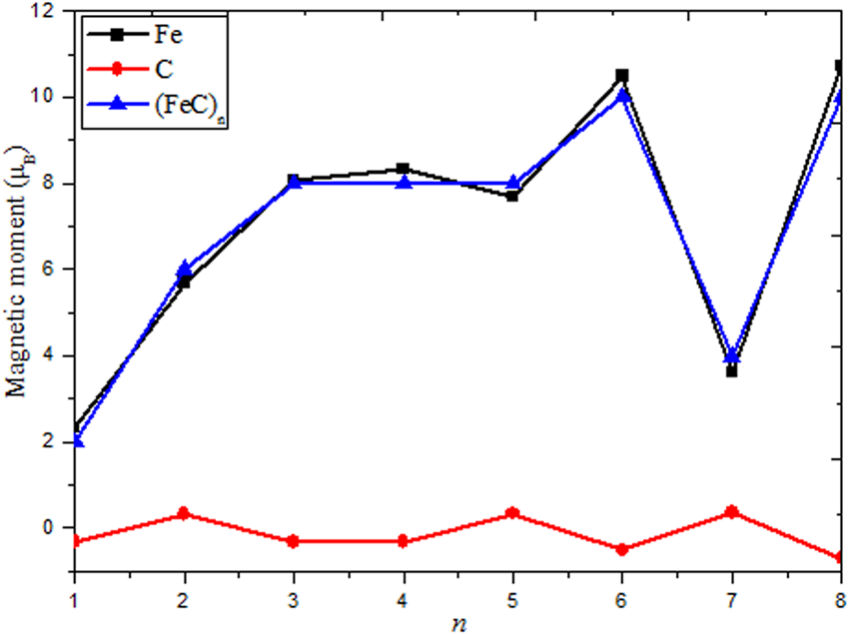
Size dependence of the local and total spin magnetic moments for the lowest energy structures of (FeC)_n_ (n = 1–8) clusters.

The magnetic properties of TM-doped clusters have been a subject of intense research owing to changed magnetic properties for the small size cluster compared to its bulk counterparts. So, detailed analysis of the on-site local and total spin magnetic moments and electron transfer are performed in current work. The local magnetic moment (*μ*_s_), total spin magnetic moment (Σ*μ*_s_) and natural populations (in unit of *e*) of the Fe and C atoms are summarized in Table 1 and Fig. 4. From Fig. 4, the local magnetic moment of Fe atom and total spin magnetic moment increase dramatically as the size changes from 1 to 3; when n = 3–5, the values almost keep constant. Starting from n >5, the local magnetic moment of Fe atom and total spin magnetic moments exhibit distinct odd-even oscillatory. However, the local magnetic moments of C atoms keep small fluctuation. In conclusion, the total spin magnetic moments are mainly located on the iron atoms site. Carbon atoms have little influence on the total spin magnetic moments. From Table 1, it can be easily inferred that the local magnetic moments mainly come from Fe-*d* states, Fe-*s* and Fe-*p* states only provide weak contribution; while the 2*p* state bring the biggest effect for C atoms. Additionally, the atomic chargers of the Fe atom possess positive charges from 0.032 to 2.243 *e*. It means that Fe acts as electron donor in all (FeC)_n_ clusters. This result may be caused by the electronegativity of Fe (1.83) is much bigger than C (2.55), which results in Fe has a stronger ability to lose electron^49^. It is well known that the configuration of valence electrons is 2*s* ^2^2*p* ^2^ for free C atom. The introduction of a carbon atom in iron clusters can undoubtedly change configuration of valence electrons. From Table 1, we find that the 2*p* state gains some amount of electrons. However, the 2s state always loses some amount of electrons. This implies that there is the internal electron transfer in C atom. In general, the internal electron transfer in C atom and the charge transfer between C and Fe atoms should be major reasons for the changes of the magnetic moments of (FeC)_n_ (n = 1–8) clusters.

**Table 1:** The local magnetic moment (*μ*_s_), total magnetic moment (|Σ*μ*_s_|), and natural populations (in unit of *e*) of the Fe and C atoms of (FeC)_n_ (n = 1–8) clusters for the lowest energy structures.

Clusters	Fe					C						Σ*μ*_s_
	*d*	*s*	*p*	*μ*_s_	Q_Fe_	*s*	charge	*p*	charge	*μ*_s_	Q_C_	
FeC	1.66	0.63	0.04	2.33	0.032	0.01	1.87	−0.34	2.14	−0.33	−0.032	2
(FeC)_2_	5.75	−0.05	−0.03	5.69	0.879	0.03	1.37	0.29	3.04	0.32	−0.879	6
(FeC)_3_	7.70	0.26	0.11	8.07	1.057	0.02	1.44	−0.34	2.88	−0.32	−1.057	8
(FeC)_4_	7.99	0.18	0.23	8.40	1.017	0.04	1.39	−0.39	2.85	−0.35	−1.017	8
(FeC)_5_	4.16	0.08	0.08	4.32	1.428	0.03	1.17	−0.37	3.10	−0.34	−1.428	8
(FeC)_6_	9.88	0.32	0.28	10.48	0.674	0.02	1.32	−0.62	2.78	−0.60	−0.674	10
(FeC)_7_	7.84	0.05	−0.06	7.86	2.243	0.05	1.24	0.08	2.80	0.17	−2.243	4
(FeC)_8_	10.32	0.24	0.16	10.72	0.823	0.04	1.24	−0.76	2.86	−0.72	−0.823	10

**Figure 5:**
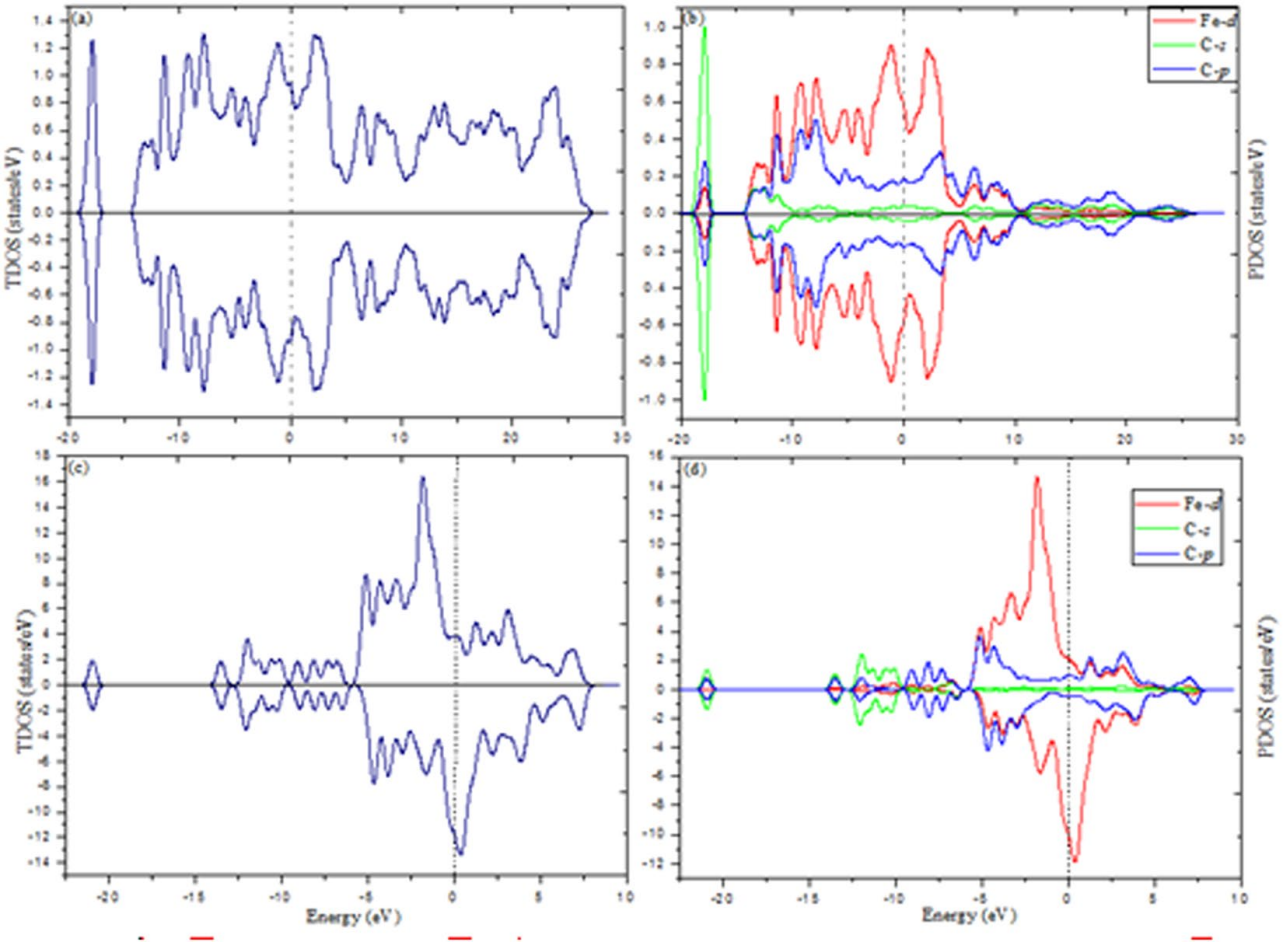
Calculated TDOS and PDOS of FeC [(a) and (b)], (FeC)_8_ [(c) and (d)] clusters.

For gaining further insight for magnetic properties, we also calculated the total density of states (TDOS) and partial density of states (PDOS) of FeC and (FeC)_8_ clusters as shown in Fig. 5. The cluster Fermi level is presented as a dashed vertical line and shifted to zero. Spin-up (alpha) and spin-down (beta) densities are given in each case. The relative shift between the spin-up and spin-down bands indicate the degree of spin exchange splitting. Generally, the larger the spin exchange splitting of DOS, the larger the magnetic moment of cluster. The present drawing are good agreement with magnetic moments of 2 *μ*_B_ and 10 *μ*_B_ for FeC and (FeC)_8_ clusters, respectively. We can see from this figure that the total spin magnetic moments mainly dominated by Fe-*d* states, while C-*s* and C-*p* states make a small contribution. This result is in agreement with the analysis of local and total spin magnetic moments in Table S1 (see SI). Ultimately, around the Fermi level the Fe-*d* is intensively overlapping not only with C-*s* electrons, but also with C-*p*, indicating that strong *sp*-*d* hybridization occurs. For FeC and (FeC)_8_ clusters, the C-*s* a slight bump stays around the Fermi level in the PDOS, but a peak is far from the Fermi level standing at −18 eV and −12 eV, respectively. Analyses indicate that the C-*s* electrons do not participate in forming the bonds and can be treated as core electrons.

### Magnetic property of (FeC)_8_TM (TM = V, Cr, Mn and Co)

**Figure 6:**
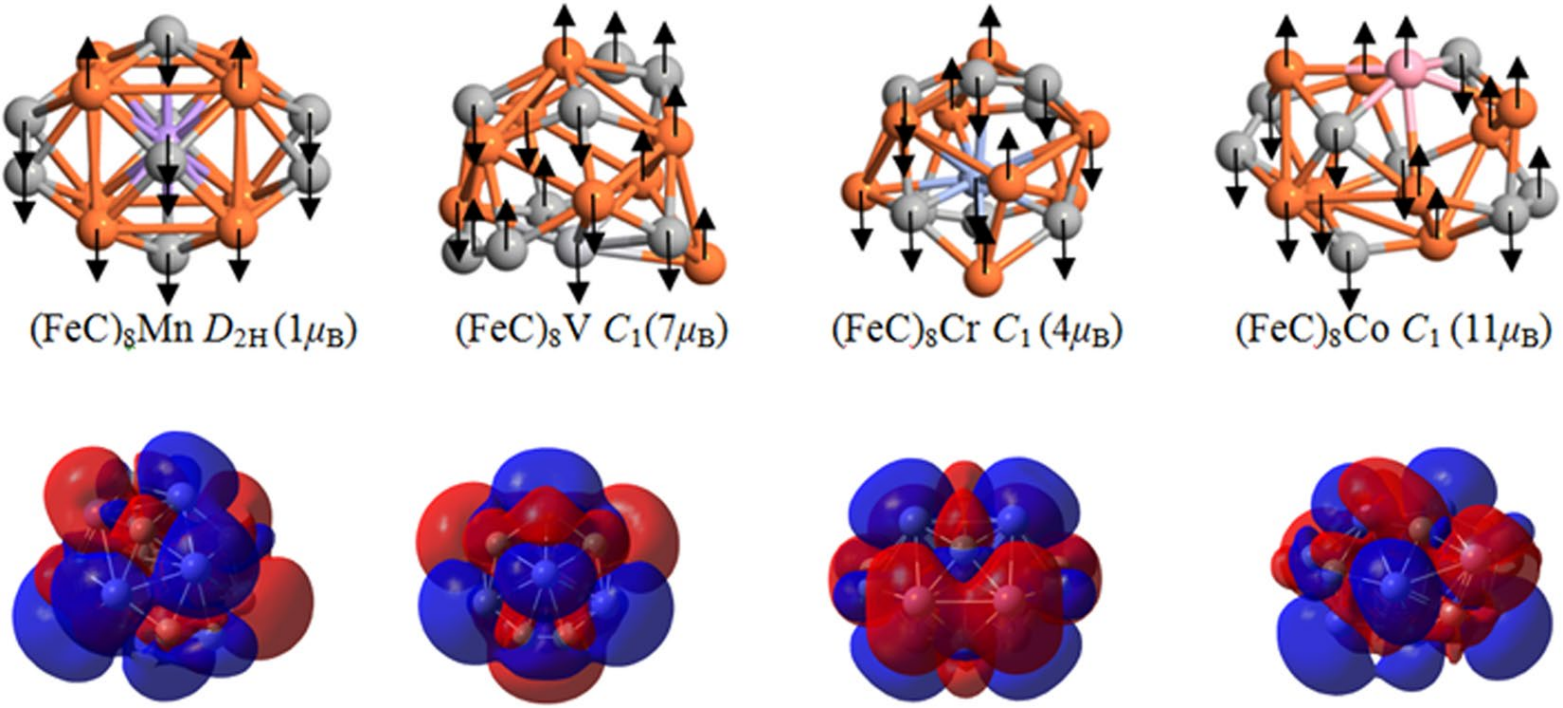
The lowest energy structures and spin density for (FeC)_8_V, (FeC)_8_Cr, (FeC)_8_Mn and (FeC)_8_Co cluster, respectively. Values in parentheses indicate the total spin magnetic moment.

As mentioned above, the lowest energy structure of (FeC)_8_ cluster has the icosioctahedron structure with a total spin magnetic moment of 10 *μ*_B_ and a mean spin moment per Fe atom of 1.34 *μ*_B_. So, this cluster can be chose as high-performance magnetic materials to explore a great variety. As we know that doping is a most efficient method to modify the electronic configuration and properties of a cluster. So, the introduction of a doped atom in (FeC)_8_ clusters, such as a TM (TM = V, Cr, Mn and Co) atom in our work, can undoubtedly change the clusters’ magnetic properties. In order to understand the magnetic behavior, the ground state should be determined firstly. Here, four different methods are investigated and summarized as follows: (1) a TM atom substitutes one Fe or C atom. (2) a TM atom is doped into the center of the cage. (3) a TM atom bridges over a Fe-C bond. (4) the structure prediction method-CALYPSO. Compared with the different methods, the lowest energy structure of (FeC)_8_TM (TM = C, Cr, Mn and V) can be determined based on the CALYPSO structure prediction method combined with density functional theory. The obtained lowest energy structures are shown in Fig. 6. Optimized structures show that (FeC)_8_Mn cluster with the same *D*_2H_ point symmetry structure retain the overall shape of the corresponding initial (FeC)_8_ cluster. Nevertheless, *C*_1_, *C*_s_ and *C*_1_ is found to be the most stable structure for (FeC)_8_V, (FeC)_8_Cr and (FeC)_8_Co cluster, respectively. Importantly, the V and Co atoms prefer to locate on the surface positions, while the Mn and Cr atoms fall into the center of the doped clusters. To further understand the magnetic behavior of doped systems, the local magnetic moment (*μ*_s_) and natural populations (in unit of *e*) of the Fe, C and TM (TM = V, Cr, Mn and Co) atoms are also calculated and presented in Table S2 (see SI). Comparing the spin orientation of different systems, in (FeC)_8_Co cluster, the moment of the Co atom align in parallel to those of the surface Fe atoms of (FeC)_8_ cluster. The other (FeC)_8_TM (TM = V, Cr and Mn) clusters are ferromagnetic ordering, where the moments of the V, Cr and Mn atom align in opposite directions. Following the order of 3*d* atom in the Periodic Table, the local magnetic moments decreases sharply and has a minimum (0.08 *μ*_B_) for Mn cluster. After this minimum, it raises sharply with a maximum (1.34 *μ*_B_) for Co cluster. Secondly, under considering spin orientation, the mean spin moment per Fe atoms decrease from 1.34 *μ*_B_ to 0.15 *μ*_B_ in (FeC)_8_Mn cluster; however, the average spin moment per Fe atom (1.26 *μ*_B_) of (FeC)_8_Co cluster is close to the origin values (1.34 *μ*_B_) in initial (FeC)_8_ cluster. So, several kinds of different magnetic behaviors result in the total spin magnetic moment enhancement with doped Co and reduction with doped V, Cr and Mn as compared to (FeC)_8_ cluster. At last, as seen from Table S2, the values of populations for the Fe atom are positive, indicating that charges are transferee from Fe atom to the C atom. Compared the values of populations of V, Cr, Mn and Co atoms, about 0.013*e* electron transferring from Co to C atom is observed in (FeC)_8_Co cluster; nevertheless, in the other three doped systems, the charges are transferred from Fe to V, Cr and Mn atoms, respectively.

In order to explore the electronic properties of the doped systems, the spin density, TDOS and PDOS are calculated and present in Fig. 6 and Figure S1 (see SI). For the spin density, there exist almost same area between blue and red in (FeC)_8_Mn cluster. It results in a very low total spin magnetic moment (1 *μ*_B_). For the DOS and PDOS, the primary features are concluded: (i) as for (FeC)_8_Mn with a total spin magnetic moment of 1 *μ*_B_, the PDOS of two spin tunnels are almost equal to each other, but for (FeC)_8_V, (FeC)_8_Cr and (FeC)_8_Co with total spin magnetic moment of 7 *μ*_B_, 4 *μ*_B_ and 11 *μ*_B_, the imbalance between two spin tunnels appear again. The larger the spin exchange splitting of TDOS corresponds to the larger total spin magnetic moments. (ii) the TDOS and Fe-*d* PDOS are almost overlapping each other, implying that the cluster properties are mostly dominated by the Fe-*d* electrons. (iii) For the PDOS of (FeC)_8_V and (FeC)_8_Cr clusters, a peak of V-*p* or Cr-*p* component are found far from the Fermi level, respectively. This implies that V-*p* or Cr-*p* electrons do not participate in forming the bonds and can be treated as core electrons.

## Conclusions

To summarize, we have performed a systematic, in–depth study of the structural, electronic and magnetic properties of (FeC)_n_ (n = 1–8) and (FeC)_8_TM (TM = C, Cr, Mn and V) clusters using CALYPSO search method combined with density functional theory. The results are summarized as follows:

(i) The planar cluster is the most stable geometries of (FeC)_n_ (n = 1–2) clusters. From n = 3, the most stable structures are all the distorted cage structures with the Fe and C atom at the vertex, except for (FeC)_5_ cluster.
(ii) Maximum peaks were observed for (FeC)_n_ clusters at n = 2, 4 and 6 on the size-dependence of second-order energy difference, implying that these clusters possess relatively higher stability than other-sized (FeC)_1,3,5,7,8_ clusters. Due to the larger HOMO-LUMO energy gaps and the characteristics of double carbon-carbon bond, (FeC)_2_ cluster has relative higher stability than other-sized (FeC)_n_ clusters.
(iii) The total spin magnetic moments are mainly contributed by the Fe atoms and partly arise from the C component. The local magnetic moments mainly come from Fe-*d* and C-2*p* states. The charges transfer from Fe atom to C atom.
(iv) For (FeC)_8_TM (TM = V, Cr, Mn and Co) clusters, the V and Co atoms prefer to locate on the surface positions, while the Mn and Cr atoms fall into the center of the doped clusters. Different magnetic behaviors result in the total spin magnetic moment enhancement with doped Co and reduction with doped V, Cr and Mn atoms. Charges tend to transfer from Fe and TM (TM = V, Cr and Mn) to C atoms. However, in (FeC)_8_Co clusters, the charges transfer from Fe to C and Co atom. At last, the properties of doped cluster are mostly dominated by the Fe-*d* electrons. And, V-*p* or Cr-*p* electrons do not participate in forming the bonds and can be treated as core electrons.

## Methods

Based on globally minimizing potential energy surfaces, the structure search is evaluated by DFT calculations through a generalized version of a particle swarm optimization (PSO) algorithm specific for cluster structure prediction, as implemented in the CALYPSO package. PSO algorithm is a global optimization algorithm based on the group search, which was proposed by Eberhart and Kennedy at 1995^50,51^. As a high efficient multiple target algorithm, it has been applied to system identification and training of neural network. Recently, Ma *et al*. have developed a CALYPSO methodology, which is the first application of PSO algorithm into extended systems. The CALYPSO method can efficiently explore the multidimensional potential energy surfaces at given external conditions (e.g., pressure) and requires only known information of chemical compositions to predict the stable structure. In the process of search, a sequence of 50 generations of structural candidates is followed to achieve convergence of the search. Each generation contain 20 structures, 70% of which are generated by PSO. The others are new and will be generated randomly. So, we can achieve 1000 structurally different low-lying isomers. Subsequently, the top 50 low-lying isomers are considered as candidates for the lowest-lying structures. These candidates are further re-optimized using the PBE functional with the 6–311 + G* basis set, implemented in the Gaussian09 package^52–55^. During the re-optimization for each cluster, the effect of the spin multiplicity is taken into account; and vibrational frequency calculations are used to verify the nature of real local minima.

## Electronic supplementary material

Supporting Information
