# Supplementary material for: First-principle study of structural, electronic and magnetic properties of (FeC)n (n = 1–8) and (FeC)8TM (TM = V, Cr, Mn and Co) clusters
Source: Sci Rep. 2017 Dec 13;7:17516. doi: 10.1038/s41598-017-17834-9 (PMC5727526; doi:10.1038/s41598-017-17834-9)
Supplement: Supplementary file 1 — Supporting Information [file 41598_2017_17834_MOESM1_ESM.doc]

**Supporting Information**

**First-principle study of structural, electronic and magnetic properties of (FeC)_n_ (n=1-8) and (FeC)_8_TM (TM=V, Cr, Mn and Co) clusters**

Cheng-Gang Li ^a, b^, Jie Zhang ^a^, Wu-Qin Zhang ^a^, Ya-Nan Tang ^a^, Bao-Zeng Ren ^b^, Yan-Fei Hu c [[1]](#footnote-2)*

*^a^* *College of Physics and Electronic Engineering, Quantum Materials Research Center, Zhengzhou Normal University, Zhengzhou 450044, China*

*^b^ School of Chemical Engineering and Energy, Zhengzhou University, Zhengzhou 450001, China*

*^c^ School of Physics and Electronic Engineering, Sichuan University of Science ＆ Engineering,*

*Zigong 643000, China*

^*^Correspondence to: Yan-Fei Hu, School of Physics and Electronic Engineering, Sichuan University of Science ＆ Engineering, Zigong 643000, China. E-mail address: [yanfei_­­hu1982@suse.edu.cn](mailto:huyanfei1982@126.com)

Cartesian cordialities for the lowest-energy structures and low-lying isomers are listed in the paper.

**FeC Cluster**

Fe 0.00000000 0.00000000 0.29314500

C 0.00000000 0.00000000 -1.27029700

**(FeC)_2_ Clusters**

2a

Fe 0.00000000 1.13242400 0.00000000

Fe -0.64986700 -1.12257900 0.00000000

C 1.04530000 -0.57220900 0.00000000

C 1.77078900 0.52954500 0.00000000

2b

Fe 0.00000000 1.01344600 0.00000000

Fe -0.65399200 -1.01823800 0.00000000

C 1.06592800 -0.55743500 0.00000000

C 1.76803900 0.57820000 0.00000000

2c

Fe 0.00000000 1.06056000 0.00000000

Fe -0.70397400 -1.04952500 0.00000000

C 1.19289600 -0.58538700 0.00000000

C 1.85765900 0.53756600 0.00000000

2d

Fe 0.00000000 0.00000000 1.72618800

Fe 0.00000000 0.00000000 -1.72618800

C 0.00000000 0.69341700 0.00000000

C 0.00000000 -0.69341700 0.00000000

**(FeC)_3_ Clusters**

3a

Fe 0.38480200 1.25501900 -0.06592800

Fe 0.89521500 -0.93788300 -0.46823900

Fe -1.32612000 -0.45971300 0.14302600

C 1.33344900 0.05862600 1.20023900

C 0.07892000 -0.38423800 1.35319900

C -1.21259200 0.94344600 -0.85849700

3b

Fe -0.49341900 -0.30094600 1.40194900

Fe 1.20051000 0.25730700 0.00000000

Fe -0.49341900 -0.30094600 -1.40194900

C -0.49341900 1.40513200 0.66704300

C -0.49341900 1.40513200 -0.66704300

C 0.06092900 -1.31705600 0.00000000

3c

Fe -0.85475800 -1.01202200 -0.06862600

Fe -0.47354700 1.14461700 -0.47024300

Fe 1.41223200 -0.02239100 0.11830900

C -1.20734100 0.34733200 1.26724900

C 0.12597500 0.42471900 1.37161600

C 0.71768000 -1.24960000 -0.81644200

3d

Fe 0.00000000 1.00898200 0.00000000

Fe 1.61624300 -0.50638700 0.00000000

C -1.65672300 0.00386100 0.00000000

Fe -0.47006000 -1.38147400 0.00000000

C -1.34000900 2.48114800 0.00000000

C -1.97006200 1.32346800 0.00000000

**(FeC)_4_ Clusters**

4a

Fe -0.12795900 0.78262200 1.16194200

Fe -1.51812400 -0.85527500 0.00000000

Fe 1.24730200 -0.90074200 0.00000000

Fe -0.12795900 0.78262200 -1.16194200

C 0.89612100 2.03848100 0.00000000

C -0.12795900 -1.07651900 1.14603200

C -0.12795900 -1.07651900 -1.14603200

C 1.64233800 0.94124200 0.00000000

4b

Fe 0.49380600 0.13728200 1.22959200

Fe -0.89618600 1.22875400 -0.49942300

Fe -1.19095900 -1.05664600 -0.17890700

Fe 1.13957700 -0.62000400 -0.79853900

C 0.89629800 1.61633300 -0.36726600

C 0.42003800 -1.60109200 0.49459400

C 1.97595500 0.93125800 -0.05365400

C -1.32598300 0.39949500 0.99786000

4c

Fe 1.20223900 -1.04218300 0.00000000

Fe -0.17644400 0.82733200 1.21639600

Fe -0.17644400 0.82733200 -1.21639600

Fe -1.46082800 -0.70308200 0.00000000

C -0.17644400 -0.97879800 -1.28599600

C 1.05532600 1.66878600 0.00000000

C -0.17644400 -0.97879800 1.28599600

C 1.94729300 0.68141700 0.00000000

4d

Fe 0.34013000 -0.91893900 1.53835700

Fe 0.27911700 1.94213300 0.00000000

Fe -1.21995100 0.25642600 0.00000000

Fe 0.34013000 -0.91893900 -1.53835700

C 0.34013000 0.78773900 -1.30207200

C -0.51042800 -1.52395600 0.00000000

C 0.95931800 -1.61447400 0.00000000

C 0.34013000 0.78773900 1.30207200

**(FeC)_5_ Clusters**

5a

Fe -0.34670300 2.99990600 0.00000000

C 1.29366400 2.02627800 0.00000000

C 1.92789800 0.88513100 0.00000000

C -1.70166800 1.74841500 0.00000000

C -2.07354800 0.46807200 0.00000000

C -1.63073500 -0.81218300 0.00000000

Fe 1.63923600 -1.01741700 0.00000000

Fe -0.09503700 0.62275800 0.00000000

Fe -0.34670300 -1.80059000 1.07444300

Fe -0.34670300 -1.80059000 -1.07444300

5b

Fe -0.33424000 2.96457100 0.00000000

C 1.31483400 2.02821700 0.00000000

C 1.85183500 0.83556900 0.00000000

C -1.76389900 1.75333400 0.00000000

C -2.06298500 0.46368000 0.00000000

C -1.58879100 -0.81713900 0.00000000

Fe 1.63109400 -1.04923800 0.00000000

Fe -0.10937300 0.58187000 0.00000000

Fe -0.33424000 -1.74056300 1.09911100

Fe -0.33424000 -1.74056300 -1.09911100

5c

Fe -0.32615900 3.01757800 0.00000000

C 1.27673200 2.01221200 0.00000000

C 1.82314400 0.81213500 0.00000000

C -1.70041900 1.74829600 0.00000000

C -2.05744200 0.46695900 0.00000000

C -1.57060300 -0.81012700 0.00000000

Fe 1.60337400 -1.04892700 0.00000000

Fe -0.11060700 0.56137100 0.00000000

Fe -0.32615900 -1.75302700 1.09352200

Fe -0.32615900 -1.75302700 -1.09352200

5d

Fe 1.93868200 0.92931200 -0.08407700

Fe 0.29305600 -0.27282900 1.25072800

Fe 1.11477700 -1.28119300 -0.75906700

Fe -0.70259200 1.06922400 -0.67998600

Fe -2.32517000 -0.40192900 0.16227500

C -0.75301800 -0.80884700 -1.02949100

C -2.28898900 1.31817800 -0.00861500

C 2.02849800 -0.67206400 0.71029300

C 0.37984600 1.49826000 0.63478200

C -0.74759700 -1.52006600 0.17024500

**(FeC)_6_ Clusters**

6a

Fe -0.08281100 1.19656100 1.45977100

Fe 0.08281100 -1.19656100 1.45977100

Fe 1.34870300 1.33461200 -0.50782800

Fe 1.08074800 -1.15515300 -0.72638000

Fe -1.08074800 1.15515300 -0.72638000

Fe -1.34870300 -1.33461200 -0.50782800

C 0.52945200 0.41285100 -1.91503800

C -0.52945200 -0.41285100 -1.91503800

C 0.02890600 2.35804200 0.10645700

C 1.34870300 0.02607700 0.83114000

C -1.34870300 -0.02607700 0.83114000

C -0.02890600 -2.35804200 0.10645700

6b

Fe -1.15546900 -0.93813700 -1.11618300

Fe 1.11863200 -1.34179000 -0.69602900

Fe -1.60895700 -0.25898900 1.15879700

Fe 0.88570700 -0.41791700 1.50019000

Fe -0.87433400 1.47614800 -0.68353100

Fe 1.63291600 1.10648200 -0.45255900

C -0.41079900 1.13547900 1.57313200

C 0.42875500 1.83129800 0.80745800

C -2.26914400 0.33605200 -0.40883300

C -0.34924000 -1.53758500 0.48017100

C 0.31473500 0.19984800 -1.52003000

C 2.29222200 -0.34354800 0.32179700

6c

Fe 0.09371900 1.18305000 1.45986500

Fe -0.09371900 -1.18305000 1.45986500

Fe 0.99954000 1.16077000 -0.73300100

Fe 1.32768000 -1.31637500 -0.50542600

Fe -1.32768000 1.31637500 -0.50542600

Fe -0.99954000 -1.16077000 -0.73300100

C 0.52252600 -0.43097500 -1.93784300

C -0.52252600 0.43097500 -1.93784300

C -0.09371900 2.39590400 0.16801900

C 1.37047400 -0.00538600 0.81026100

C -1.37047400 0.00538600 0.81026100

C 0.09371900 -2.39590400 0.16801900

6d

Fe 1.80856100 -0.48441800 -0.92054300

Fe -1.66919200 -0.64774900 0.92123900

Fe -0.72807800 -0.74385300 -1.29956100

Fe 1.00378700 1.54045700 0.31559300

Fe -1.27763800 1.39228300 -0.37489600

Fe 0.82925800 -0.61201800 1.36579100

C 0.32922600 0.84915000 -1.25139600

C -0.37174400 -1.90824800 0.38239000

C 2.25895900 0.27698000 0.62793500

C -0.45795500 0.81231000 1.25260500

C 0.70470900 -1.92154400 -0.42711500

C -2.31889100 -0.03568400 -0.61745100

**(FeC)_7_ Clusters**

7a

Fe -0.05913600 0.18050900 -1.25236500

Fe 2.19710800 -0.84722700 -0.55490200

Fe -1.19298100 0.98470500 0.87656300

Fe 1.26582600 0.34213400 1.33079900

Fe -2.05389800 -1.00966300 -0.35386200

Fe 0.13346400 -1.67984100 0.59533200

Fe 0.47073600 2.34128900 -0.31438600

C 1.63616800 0.92653300 -0.43909500

C -1.27589500 1.92312600 -1.00621000

C 0.77547600 -1.79883200 -1.33797900

C -1.44901000 -0.97265300 1.53807600

C -0.36765500 -0.39784400 2.10412000

C -0.56057800 -1.87788800 -1.24760200

C -2.05668600 0.84596800 -1.02908900

7b

Fe -1.38605500 0.88244900 -1.09896100

Fe 1.48412400 -1.28588300 0.81896800

Fe 2.04715000 0.50720800 -0.69589900

Fe 0.18909500 -0.97928200 -1.14985100

Fe -1.92362500 -1.19322600 0.16432800

Fe -0.35800300 0.09894700 1.58325400

Fe 0.35269000 1.95002400 0.40096200

C -1.65890400 -0.89520000 -1.52567100

C -2.22121400 0.52757100 0.83610700

C -1.50094100 1.67005400 0.83052300

C 0.42302000 1.10433400 -1.28799000

C 2.03046500 -1.26171400 -0.89719800

C 1.48364200 0.58826100 1.11827300

C -0.31269800 -1.64766500 0.82715000

7c

Fe -1.27538100 0.82846300 -1.11527100

Fe 0.35398400 1.94393800 0.34842100

Fe 2.06551500 0.46976100 -0.67313000

Fe -1.93866900 -1.14567000 0.12297000

Fe 1.43585700 -1.26227100 0.91257600

Fe 0.21588400 -1.03836200 -1.14185100

Fe -0.45311500 0.15378200 1.59486300

C 0.49631000 1.12479600 -1.33359400

C -2.25991800 0.58433700 0.72779600

C 1.39891400 0.59880500 1.14440700

C -1.60530700 -0.89125200 -1.58346400

C -0.36095200 -1.59992400 0.87266800

C 2.06121500 -1.29689700 -0.76122200

C -1.48125000 1.69835300 0.72290700

7d

Fe 0.05178000 0.09084400 -0.10086800

Fe 0.27158800 2.44949700 -0.27619800

Fe -0.53008500 -2.16001100 0.32104900

Fe -2.12917200 0.43504300 1.10822500

Fe 2.10149000 -0.73690400 -1.16259100

Fe 2.05640100 -0.07896400 1.19117600

Fe -2.18738400 0.07962800 -1.15710900

C -1.34310400 1.49704500 -0.22141800

C -0.93747700 -1.29424200 -1.44858800

C 1.86753600 1.82498300 0.54058000

C 0.46078500 -1.05414200 1.68057900

C -0.85732500 -0.79177100 1.72191500

C 2.01606500 1.04437100 -0.55908600

C 0.37684700 -1.56915200 -1.38327800

**(FeC)_8_ Clusters**

8a

Fe -1.25655900 0.00000000 1.41314500

Fe 1.28281700 1.95463700 0.00000000

Fe 1.25655900 0.00000000 1.41314500

Fe -1.28281700 1.95463700 0.00000000

Fe -1.28281700 -1.95463700 0.00000000

Fe 1.25655900 0.00000000 -1.41314500

Fe 1.28281700 -1.95463700 0.00000000

Fe -1.25655900 0.00000000 -1.41314500

C 2.64135600 0.67342700 0.00000000

C -2.64135600 -0.67342700 0.00000000

C 0.00000000 1.48102800 1.26603200

C 0.00000000 -1.48102800 1.26603200

C 0.00000000 1.48102800 -1.26603200

C 0.00000000 -1.48102800 -1.26603200

C -2.64135600 0.67342700 0.00000000

C 2.64135600 -0.67342700 0.00000000

8b

Fe 0.57823900 -1.07654100 1.48614000

Fe -0.57858500 1.07848700 -1.48954800

Fe 0.96977600 2.08955900 0.12669500

Fe -0.95080800 -2.10856300 -0.13407300

Fe 2.39745600 0.08873700 0.05802000

Fe -2.38165600 -0.07692900 -0.10809600

Fe 0.80773400 -0.91847800 -1.42536000

Fe -0.81251100 0.93131100 1.48831700

C -0.90623200 2.51690500 0.07459200

C 0.87595400 -2.53007900 -0.08453100

C -2.01293500 1.79012800 -0.06885900

C 1.97251400 -1.79337900 0.11255500

C 1.37498400 0.93954500 -1.30512700

C -1.35731900 -0.96966200 1.22899300

C 1.10944700 0.76580700 1.41684900

C -1.18487800 -0.75212500 -1.38355200

8c

Fe 0.94452300 -0.78517600 1.39496800

Fe -2.30920100 -0.75657200 0.00000000

Fe -0.94452300 0.78517600 1.39496800

Fe -0.27810600 -2.31124100 0.00000000

Fe 2.30920100 0.75657200 0.00000000

Fe -0.94452300 0.78517600 -1.39496800

Fe 0.27810600 2.31124100 0.00000000

Fe 0.94452300 -0.78517600 -1.39496800

C -2.52672900 1.14593900 0.00000000

C 2.52672900 -1.14593900 0.00000000

C -0.94452300 -1.12748000 1.31782600

C 0.94452300 1.12748000 1.31782600

C -0.94452300 -1.12748000 -1.31782600

C 0.94452300 1.12748000 -1.31782600

C 1.64586500 -2.15175900 0.00000000

C -1.64586500 2.15175900 0.00000000

8d

Fe -0.94257600 -0.79016200 1.39664500

Fe 0.94257600 0.79016200 -1.39664500

Fe 2.22606400 -0.68159900 0.00000000

Fe -2.22606400 0.68159900 0.00000000

Fe 0.33622400 -2.39968500 0.00000000

Fe -0.33622400 2.39968500 0.00000000

Fe -0.94257600 -0.79016200 -1.39664500

Fe 0.94257600 0.79016200 1.39664500

C 2.41013900 1.24005000 0.00000000

C -2.41013900 -1.24005000 0.00000000

C 1.57392600 2.28328700 0.00000000

C -1.57392600 -2.28328700 0.00000000

C 0.94257600 -1.12639800 -1.32135800

C -0.94257600 1.12639800 1.32135800

C 0.94257600 -1.12639800 1.32135800

C -0.94257600 1.12639800 -1.32135800

**(FeC)_8_Co Clusters**

Fe -1.24915300 1.66113900 0.71627400

Fe 0.41503400 0.00323400 -1.82821900

Fe 0.98769200 1.86030200 -0.39148400

Fe -2.04049700 0.51894900 -1.26101200

Fe -0.41538600 -0.44871900 1.84147600

Fe 2.30709200 -1.22515100 -0.51284100

Fe 2.02121800 0.38211500 1.37117800

Fe -2.12045300 -1.49812900 0.18860000

C 2.24792700 0.60484200 -1.42057000

C -2.27865100 0.03318100 1.51445200

C -0.74792800 1.77243100 -1.08006000

C 0.41234900 1.30896700 1.38855000

C -1.20627200 -1.08255200 -1.40250800

C 1.31629900 -1.27555300 1.20432400

C -2.91501300 0.50662600 0.42373300

C 2.86976100 0.90184600 -0.27219900

Co 0.15796100 -1.82281200 -0.19842900

**(FeC)_8_Mn Clusters**

Fe -1.40319400 0.00000000 1.72250200

Fe 1.29491100 1.95261500 0.00000000

Fe 1.40319400 0.00000000 1.72250200

Fe -1.29491100 1.95261500 0.00000000

Fe -1.29491100 -1.95261500 0.00000000

Fe 1.40319400 0.00000000 -1.72250200

Fe 1.29491100 -1.95261500 0.00000000

Fe -1.40319400 0.00000000 -1.72250200

C 2.64241200 0.67743700 0.00000000

C -2.64241200 -0.67743700 0.00000000

C 0.00000000 1.46962200 1.40925000

C 0.00000000 -1.46962200 1.40925000

C 0.00000000 1.46962200 -1.40925000

C 0.00000000 -1.46962200 -1.40925000

C -2.64241200 0.67743700 0.00000000

C 2.64241200 -0.67743700 0.00000000

Mn 0.00000000 0.00000000 0.00000000

**(FeC)_8_V Clusters**

Fe 1.00670200 1.89002400 0.49002000

Fe 2.11184700 -1.39515900 -0.14318900

Fe 1.01252300 -0.22301300 1.78777500

Fe 1.81002500 0.62545900 -1.48641400

Fe -1.48739200 1.89052400 0.52413600

Fe -0.31328500 -1.97308400 -0.50827800

Fe -1.43012300 -0.52928600 1.22601000

Fe -2.52485300 -1.04102900 -1.00527300

C 1.08379100 -2.29974200 1.17088000

C -0.38528500 2.58846200 -0.84603000

C 2.21862800 0.39546000 0.37531800

C -0.30229100 1.16532100 1.70213500

C 0.93322500 -1.02433800 -1.57780500

C -2.49153300 0.54552600 -0.09139600

C 0.68429900 2.16327200 -1.52743700

C -0.14392400 -1.93134100 1.55075000

V -0.62621900 0.43604100 -1.19751900

**(FeC)_8_Cr Clusters**

Fe -0.13096400 -2.07728000 0.00000000

Fe 1.14703100 0.66060300 2.15890900

Fe 2.13371800 -0.97520700 0.00000000

Fe -0.98551100 -0.67652200 1.95480700

Fe -0.98551100 -0.67652200 -1.95480700

Fe -0.72712600 2.41136100 0.00000000

Fe 1.14703100 0.66060300 -2.15890900

Fe -2.04194100 0.57514100 0.00000000

C 2.33897100 1.08032900 0.65777800

C -2.10910200 -1.49382400 -0.66615200

C 0.81354300 -1.22541700 1.45335600

C 0.81354300 -1.22541700 -1.45335600

C -0.65022800 1.19466200 1.60597900

C -0.65022800 1.19466200 -1.60597900

C -2.10910200 -1.49382400 0.66615200

C 2.33897100 1.08032900 -0.65777800

Cr 0.28362000 0.32810200 0.00000000

Mayer bond order of the lowest energy structures (FeC)_n_ clusters.

(FeC)_2_

1Fe-2Fe 1.359 3C=4C 1.986

(FeC)_3_

1Fe-2Fe 1.186 1Fe-3Fe 0.740

4C-5C 1.307 2Fe-3Fe 1.151

(FeC)_4_

1Fe-2Fe 0.639 1Fe-4Fe 1.014

3C-6C 1.232 1Fe-5Fe 1.014

2Fe-4Fe 0.688 2Fe-5Fe 0.688

4Fe-5Fe 1.009

(FeC)_5_

1Fe-8Fe 0.901 2C-3C 1.140

7Fe-8Fe 0.172 4C-5C 0.754

8Fe-9Fe 0.283 5C-6C 0.616

8Fe-10Fe 0.283 7Fe-9Fe 1.263

7Fe-10Fe 1.263 9Fe-10Fe 1.841

(FeC)_6_

1Fe-2Fe 0.845 7C-8C 1.115

1Fe-3Fe 0.560 2Fe-5Fe 0.214

2Fe-6Fe 0.560 5Fe-6Fe 0.574

3Fe-4Fe 0.574 4Fe-5Fe 0.198

4Fe-6Fe 0.585 3Fe-5Fe 0.585

3Fe-6Fe 0.271 2Fe-3Fe 0.414

2Fe-4Fe 0.511 1Fe-5Fe 0.511

1Fe-6Fe 0.414 1Fe-4Fe 0.214

(FeC)_7_

1Fe-2Fe 0.551 9C-14C 1.588

1Fe-3Fe 0.344 10C-13C 1.311

1Fe-4Fe 0.247 11C-12C 1.253

1Fe-5Fe 0.162 2Fe-5Fe 0.157

1Fe-6Fe 0.118 2Fe-4Fe 0.374

2Fe-6Fe 0.165 2Fe-7Fe 0.513

3Fe-4Fe 0.453 3Fe-5Fe 0.874

3Fe-6Fe 0.453 3Fe-7Fe 0.458

4Fe-5Fe 0.105 5Fe-6Fe 0.574

4Fe-6Fe 0.209 4Fe-7Fe 1.463

5Fe-7Fe 0.178 6Fe-7Fe 0.086

(FeC)_8_

| 1Fe-2Fe | 0.163 | 9C-16C | 0.950 |
| --- | --- | --- | --- |
| 1Fe-3Fe | 0.672 | 10C-15C | 0.950 |
| 1Fe-4Fe | 0.534 | 3Fe-6Fe | 0.336 |
| 1Fe-5Fe | 0.534 | 3Fe-7Fe | 0.534 |
| 1Fe-6Fe | 0.124 | 3Fe-8Fe | 0.124 |
| 1Fe-7Fe | 0.163 | 4Fe-5Fe | 0.287 |
| 1Fe-8Fe | 0.336 | 4Fe-6Fe | 0.163 |
| 2Fe-3Fe | 0.534 | 4Fe-7Fe | 0.086 |
| 2Fe-4Fe | 0.926 | 4Fe-8Fe | 0.534 |
| 2Fe-5Fe | 0.086 | 5Fe-6Fe | 0.163 |
| 2Fe-6Fe | 0.534 | 5Fe-7Fe | 0.926 |
| 2Fe-7Fe | 0.287 | 5Fe-8Fe | 0.534 |
| 2Fe-8Fe | 0.163 | 6Fe-7Fe | 0.534 |
| 3Fe-4Fe | 0.163 | 6Fe-8Fe | 0.671 |
| 3Fe-5Fe | 0.163 | 7Fe-8Fe | 0.163 |

**Table S1** the local and total magnetic moments of per atom for the lowest energy structures and some low-lying metastable isomers of (FeC)_n_ (n=1-8) clusters.

**FeC**

1

| Atom | *μ*_s_ | Atom | *μ*_s_ | Σ*μ*_s_ |
| --- | --- | --- | --- | --- |
| Fe | -2.35 | C | 0.35 | 2.00 |

**Fe_2_C_2_**

1

| Atom | *μ*_s_ | Atom | *μ*_s_ |
| --- | --- | --- | --- |
| 1Fe | -3.02 | 3C | -0.14 |
| 2Fe | -2.69 | 4C | -0.14 |

2

| Atom | *μ*_s_ | Atom | *μ*_s_ |
| --- | --- | --- | --- |
| 1Fe | -2.11 | 3C | 0.03 |
| 2Fe | -1.91 | 4C | -0.02 |

3

| Atom | *μ*_s_ | Atom | *μ*_s_ |
| --- | --- | --- | --- |
| 1Fe | -3.46 | 3C | -0.33 |
| 2Fe | -3.94 | 4C | -0.26 |

4

| Atom | *μ*_s_ | Atom | *μ*_s_ |
| --- | --- | --- | --- |
| 1Fe | -2.89 | 3C | -0.10 |
| 2Fe | -2.89 | 4C | -0.10 |

**Fe_3_C_3_**

1

| Atom | *μ*_s_ | Atom | *μ*_s_ |
| --- | --- | --- | --- |
| 1Fe | -2.75 | 4C | 0.006 |
| 2Fe | -3.00 | 5C | -0.11 |
| 3Fe | -2.35 | 6C | 0.22 |

2

| Atom | *μ*_s_ | Atom | *μ*_s_ |
| --- | --- | --- | --- |
| 1Fe | -2.48 | 4C | -0.17 |
| 2Fe | -2.85 | 5C | -0.17 |
| 3Fe | -2.48 | 6C | 0.16 |

3

| Atom | *μ*_s_ | Atom | *μ*_s_ |
| --- | --- | --- | --- |
| 1Fe | -1.88 | 4C | -0.04 |
| 2Fe | -2.88 | 5C | -0.14 |
| 3Fe | -1.39 | 6C | 0.34 |

4

| Atom | *μ*_s_ | Atom | *μ*_s_ |
| --- | --- | --- | --- |
| 1Fe | -2.19 | 4C | -2.85 |
| 2Fe | -3.22 | 5C | 0.22 |
| 3Fe | 0.15 | 6C | -0.09 |

**Fe_4_C_4_**

1

| Atom | *μ*_s_ | Atom | *μ*_s_ |
| --- | --- | --- | --- |
| 1Fe | -2.49 | 5C | -0.02 |
| 2Fe | -1.99 | 6C | 0.25 |
| 3Fe  4Fe | -1.32  -2.49 | 7C  8C | 0.25  -0.16 |

2

| Atom | *μ*_s_ | Atom | *μ*_s_ |
| --- | --- | --- | --- |
| 1Fe | -2.00 | 5C | -0.09 |
| 2Fe | -2.01 | 6C | 0.38 |
| 3Fe  4Fe | -2.41  -2.11 | 7C  8C | 0.02  0.31 |

3

| Atom | *μ*_s_ | Atom | *μ*_s_ |
| --- | --- | --- | --- |
| 1Fe | -1.89 | 5C | 0.09 |
| 2Fe | -2.20 | 6C | 0.09 |
| 3Fe  4Fe | -2.20  2.24 | 7C  8C | 0.09  -0.22 |

4

| Atom | *μ*_s_ | Atom | *μ*_s_ |
| --- | --- | --- | --- |
| 1Fe | -0.13 | 5C | 0.12 |
| 2Fe | -1.14 | 6C | 0.04 |
| 3Fe  4Fe | -2.86  -0.13 | 7C  8C | 0.002  0.12 |

**Fe_5_C_5_**

1

| Atom | *μ*_s_ | Atom | *μ*_s_ |
| --- | --- | --- | --- |
| 1Fe | 2.51 | 6C | -0.14 |
| 2Fe | -3.03 | 7C | -0.06 |
| 3Fe  4Fe  5Fe | -1.46  -2.85  -2.85 | 8C  9C  10C | 0.01  -0.07  -0.04 |

2

| Atom | *μ*_s_ | Atom | *μ*_s_ |
| --- | --- | --- | --- |
| 1Fe | 3.18 | 6C | 0.08 |
| 2Fe | -2.41 | 7C | 0.18 |
| 3Fe  4Fe  5Fe | 1.63  -2.54  -2.54 | 8C  9C  10C | 0.15  0.13  0.12 |

3

| Atom | *μ*_s_ | Atom | *μ*_s_ |
| --- | --- | --- | --- |
| 1Fe | 3.22 | 6C | 0.11 |
| 2Fe | -3.07 | 7C | 0.06 |
| 3Fe  4Fe  5Fe | 1.44  -2.99  -2.99 | 8C  9C  10C | 0.19  0.07  -0.04 |

4

| Atom | *μ*_s_ | Atom | *μ*_s_ |
| --- | --- | --- | --- |
| 1Fe | -2.46 | 6C | -0.11 |
| 2Fe | -1.90 | 7C | 0.19 |
| 3Fe  4Fe  5Fe | -2.57  0.64  -2.12 | 8C  9C  10C | 0.17  0.17  -0.07 |

**Fe_6_C_6_**

1

| Atom | *μ*_s_ | Atom | *μ*_s_ |
| --- | --- | --- | --- |
| 1Fe | -1.78 | 7C | -0.04 |
| 2Fe | -1.78 | 8C | -0.04 |
| 3Fe  4Fe  5Fe  6Fe | -1.51  -1.96  -1.96  -1.51 | 9C  10C  11C  12C | 0.24  0.05  0.05  0.24 |

2

| Atom | *μ*_s_ | Atom | *μ*_s_ |
| --- | --- | --- | --- |
| 1Fe | -2.03 | 7C | 0.07 |
| 2Fe | -2.14 | 8C | -0.01 |
| 3Fe  4Fe  5Fe  6Fe | -1.97  -2.16  2.13  1.93 | 9C  10C  11C  12C | 0.05  0.08  0.03  0.03 |

3

| Atom | *μ*_s_ | Atom | *μ*_s_ |
| --- | --- | --- | --- |
| 1Fe | -1.72 | 7C | -0.03 |
| 2Fe | -1.72 | 8C | -0.03 |
| 3Fe | -2.03 | 9C | 0.28 |
| 4Fe | -0.64 | 10C | 0.16 |
| 5Fe | -0.64 | 11C | 0.16 |
| 6Fe | -2.03 | 12C | 0.28 |

4

| Atom | *μ*_s_ | Atom | *μ*_s_ |
| --- | --- | --- | --- |
| 1Fe | -1.87 | 7C | 0.06 |
| 2Fe | -2.02 | 8C | -0.01 |
| 3Fe | -0.94 | 9C | 0.19 |
| 4Fe | 1.65 | 10C | 0.01 |
| 5Fe | -1.41 | 11C | 0.09 |
| 6Fe | -1.77 | 12C | 0.03 |

**Fe_7_C_7_**

1

| Atom | *μ*_s_ | Atom | *μ*_s_ |
| --- | --- | --- | --- |
| 1Fe | -2.83 | 8C | 0.06 |
| 2Fe | 2.54 | 9C | -0.06 |
| 3Fe | -2.77 | 10C | 0.01 |
| 4Fe | 1.89 | 11C | -0.10 |
| 5Fe | -2.70 | 12C | -0.03 |
| 6Fe | 0.07 | 13C | -0.12 |
| 7Fe | -0.19 | 14C | -0.15 |

2

| Atom | *μs* | Atom | *μs* |
| --- | --- | --- | --- |
| 1Fe | 0.39 | 8C | -0.02 |
| 2Fe | -0.11 | 9C | 0.002 |
| 3Fe | -1.74 | 10C | -0.008 |
| 4Fe | -1.35 | 11C | 0.11 |
| 5Fe | 0.76 | 12C | 0.19 |
| 6Fe | -2.30 | 13C | 0.14 |
| 7Fe | -2.17 | 14C | 0.06 |

3

| Atom | *μ*_s_ | Atom | *μ*_s_ |
| --- | --- | --- | --- |
| 1Fe | 2.49 | 8C | 0.15 |
| 2Fe | -1.59 | 9C | -0.14 |
| 3Fe | -2.60 | 10C | 0.05 |
| 4Fe | -1.59 | 11C | 0.05 |
| 5Fe | -2.60 | 12C | -0.03 |
| 6Fe | 2.86 | 13C | -0.03 |
| 7Fe | -2.84 | 14C | -0.16 |

4

| Atom | *μ*_s_ | Atom | *μ*_s_ |
| --- | --- | --- | --- |
| 1Fe | -1.83 | 8C | 0.01 |
| 2Fe | -1.60 | 9C | -0.07 |
| 3Fe | -1.70 | 10C | -0.05 |
| 4Fe | 2.00 | 11C | 0.36 |
| 5Fe | 0.50 | 12C | 0.06 |
| 6Fe | -2.25 | 13C | 0.14 |
| 7Fe | -1.59 | 14C | 0.00 |

**Fe_8_C_8_**

1

| Atom | *μ*_s_ | Atom | *μ*_s_ |
| --- | --- | --- | --- |
| 1Fe | -1.92 | 9C | 0.04 |
| 2Fe | -0.76 | 10C | 0.04 |
| 3Fe | -1.92 | 11C | 0.14 |
| 4Fe | -0.76 | 12C | 0.14 |
| 5Fe | -0.76 | 13C | 0.14 |
| 6Fe | -1.92 | 14C | 0.14 |
| 7Fe | -0.76 | 15C | 0.04 |
| 8Fe | -1.92 | 16C | 0.04 |

2

| Atom | *μ*_s_ | Atom | *μ*_s_ |
| --- | --- | --- | --- |
| 1Fe | -1.81 | 9C | 0.04 |
| 2Fe | -1.53 | 10C | 0.04 |
| 3Fe | -1.81 | 11C | 0.11 |
| 4Fe | 1.93 | 12C | 0.11 |
| 5Fe | -1.53 | 13C | 0.11 |
| 6Fe | -1.81 | 14C | 0.11 |
| 7Fe | 1.93 | 15C | -0.04 |
| 8Fe | -1.81 | 16C | -0.04 |

3

| Atom | *μ*_s_ | Atom | *μ*_s_ |
| --- | --- | --- | --- |
| 1Fe | -1.55 | 9C | 0.05 |
| 2Fe | -2.24 | 10C | -0.01 |
| 3Fe | -1.23 | 11C | -0.02 |
| 4Fe | 1.82 | 12C | -0.03 |
| 5Fe | 2.20 | 13C | 0.14 |
| 6Fe | 2.09 | 14C | -0.02 |
| 7Fe | -1.45 | 15C | 0.14 |
| 8Fe | -1.93 | 16C | 0.04 |

4

| Atom | *μ*_s_ | Atom | *μ*_s_ |
| --- | --- | --- | --- |
| 1Fe | -1.82 | 9C | -0.04 |
| 2Fe | -1.82 | 10C | -0.04 |
| 3Fe | 1.93 | 11C | 0.03 |
| 4Fe | 1.93 | 12C | 0.03 |
| 5Fe | -1.52 | 13C | 0.11 |
| 6Fe | -1.52 | 14C | 0.11 |
| 7Fe | -1.82 | 15C | 0.11 |
| 8Fe | -1.82 | 16C | 0.11 |

**Table S2** The local magnetic moments and natural populations for the lowest energy structures of (FeC)_8_TM (TM=V, Cr, Mn and Co) clusters.

**Fe8C8V**  7*μ*B

| Atom | *μ*_s_ | Q | Atom | *μ*_s_ | Q |
| --- | --- | --- | --- | --- | --- |
| 1Fe | -1.87 | 0.18 | 9C | -0.09 | -0.24 |
| 2 Fe | 1.90 | 0.20 | 10 C | -0.02 | -0.11 |
| 3 Fe | -2.28 | 0.30 | 11 C | 0.01 | -0.15 |
| 4 Fe | 1.32 | 0.12 | 12 C | 0.06 | -0.10 |
| 5 Fe | 0.56 | -0.06 | 13 C | -0.05 | -0.17 |
| 6 Fe | -2.32 | 0.20 | 14 C | 0.02 | -0.20 |
| 7Fe | -2.31 | 0.13 | 15C | -0.03 | -0.18 |
| 8Fe | -2.59 | 0.54 | 16C | -0.03 | -0.27 |
|  |  |  | 17V | 0.71 | -0.18 |

**Fe_8_C_8_Cr**  4*μ*_B_

| Atom | *μ*_s_ | Q | Atom | *μ*_s_ | Q |
| --- | --- | --- | --- | --- | --- |
| 1Fe | -1.75 | 0.20 | 9C | 0.07 | -0.22 |
| 2 Fe | 2.48 | 0.52 | 10 C | 0.02 | -0.21 |
| 3 Fe | -1.66 | 0.37 | 11 C | 0.08 | -0.09 |
| 4 Fe | 1.49 | 0.05 | 12 C | 0.08 | -0.14 |
| 5 Fe | 1.95 | 0.20 | 13 C | 0.04 | -0.25 |
| 6 Fe | -2.37 | 0.61 | 14 C | 0.04 | -0.26 |
| 7Fe | -2.23 | 0.46 | 15C | 0.00 | -0.17 |
| 8Fe | -2.37 | 0.38 | 16C | 0.03 | -0.13 |
|  |  |  | 17Cr | 0.09 | -1.32 |

**Fe8C8Mn** 1*μ*B

| Atom | *μ*_s_ | Q | Atom | *μ*_s_ | Q |
| --- | --- | --- | --- | --- | --- |
| 1Fe | -2.17 | 0.48 | 9C | 0.01 | -0.21 |
| 2 Fe | 1.87 | 0.22 | 10 C | 0.01 | -0.21 |
| 3 Fe | -2.17 | 0.48 | 11 C | 0.03 | -0.16 |
| 4 Fe | 1.87 | 0.22 | 12 C | 0.03 | -0.16 |
| 5 Fe | 1.87 | 0.22 | 13 C | 0.03 | -0.16 |
| 6 Fe | -2.17 | 0.48 | 14 C | 0.03 | -0.16 |
| 7Fe | 1.87 | 0.22 | 15C | 0.01 | -0.21 |
| 8Fe | -2.17 | 0.48 | 16C | 0.01 | -0.21 |
|  |  |  | 17Mn | 0.08 | -1.34 |

**Fe_8_C_8_Co** 11*μ*_B_

| Atom | *μ*_s_ | Q | Atom | *μ*_s_ | Q |
| --- | --- | --- | --- | --- | --- |
| 1Fe | 1.54 | 0.13 | 9C | 0.02 | -0.31 |
| 2 Fe | -2.51 | 0.31 | 10 C | 0.01 | -0.30 |
| 3 Fe | -0.60 | 0.06 | 11 C | 0.01 | -0.05 |
| 4 Fe | 0.38 | -0.06 | 12 C | 0.10 | -0.08 |
| 5 Fe | -2.28 | 0.23 | 13 C | 0.11 | -0.31 |
| 6 Fe | -2.69 | 0.51 | 14 C | 0.19 | -0.27 |
| 7Fe | -1.32 | 0.07 | 15C | 0.00 | -0.20 |
| 8Fe | -2.59 | 0.48 | 16C | -0.05 | -0.24 |
|  |  |  | 17Co | -1.34 | 0.01 |

**Figure S1** Calculated TDOS and PDOS of (FeC)_8_TM (TM=V, Cr, Mn and Co) clusters.


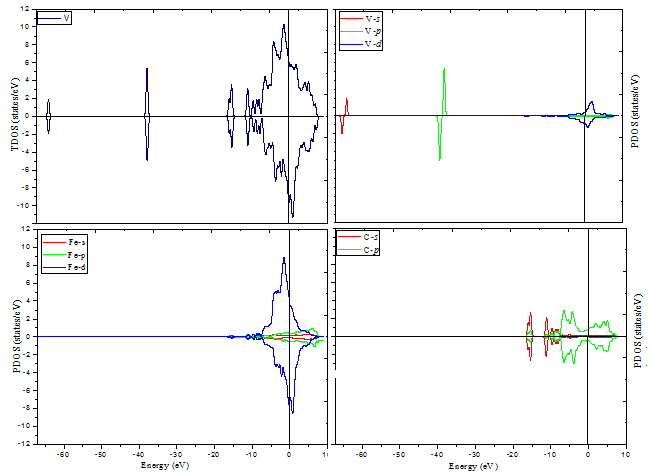


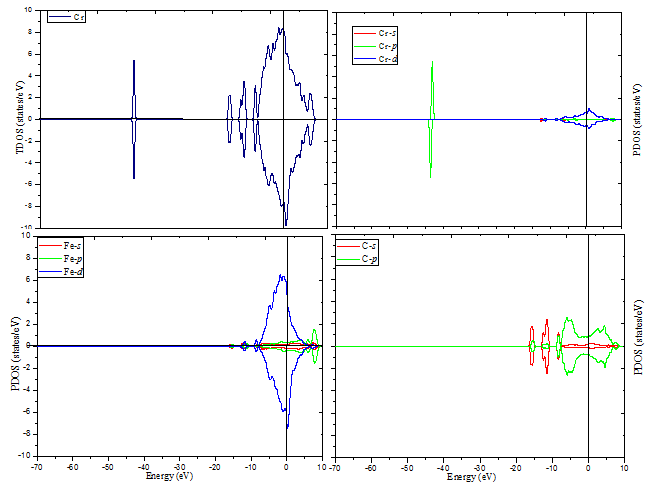


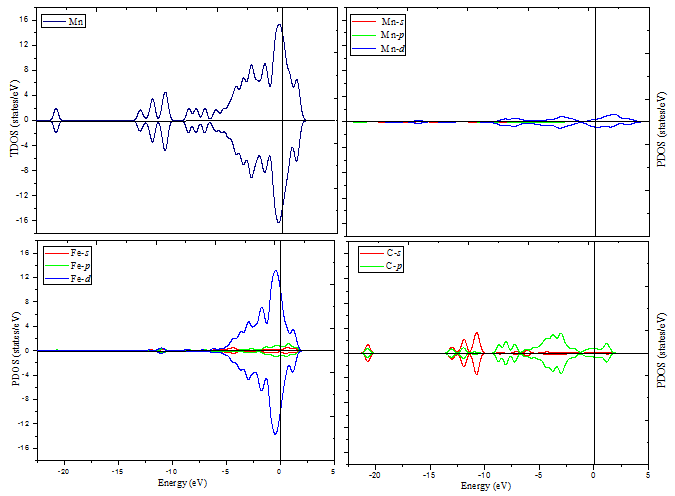


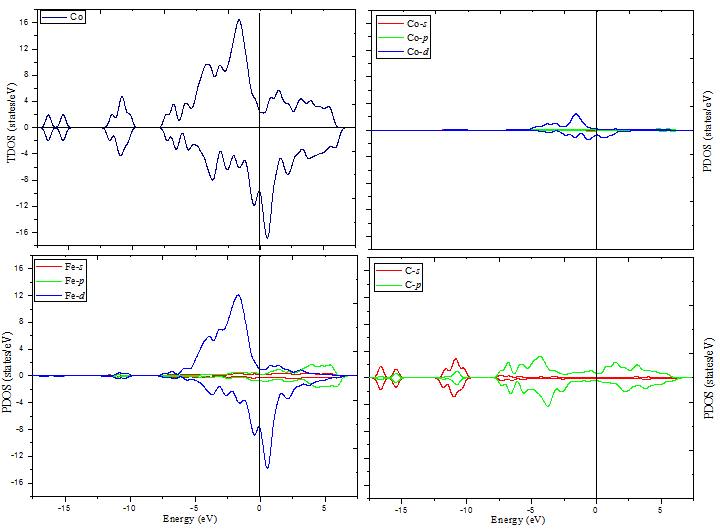


1. ^*^ Correspondence to: Yan-Fei Hu, School of Physics and Electronic Engineering, Sichuan University of Science ＆ Engineering, Zigong 643000, China. E-mail address: [yanfei_­­hu1982@suse.edu.cn](mailto:huyanfei1982@126.com) [↑](#footnote-ref-2)
